# Supplementary material for: Heterogeneity in Utilization of Optical Imaging Guided Surgery for Identifying or Preserving the Parathyroid Glands—A Meta-Narrative Review
Source: Life (Basel). 2022 Mar 8;12(3):388. doi: 10.3390/life12030388 (PMC8955594; doi:10.3390/life12030388)
Supplement: Supplementary file 1 [file life-12-00388-s001.zip › Table S1; Search string.pdf]

(((thyroid surgery) OR (thyroidectomy) OR (total thyroidectomy)) AND (autofluorescence)) OR (((thyroid surgery) OR (thyroidectomy) OR (total thyroidectomy)) AND (NIR-AF)) OR (((thyroid surgery) OR (thyroidectomy) OR (total thyroidectomy)) AND (fluorescence)) OR (((thyroid surgery) OR (thyroidectomy) OR (total thyroidectomy)) AND ((ICG) OR (indocyanine green))) OR (((thyroid surgery) OR (thyroidectomy) OR (total thyroidectomy)) AND ((LSCI) OR (LASCA) OR (laser speckle contrast imaging) OR (laser speckle contrast analysis) OR (laser speckle contrast)))
